# Mitochondrial reactive oxygen species perturb AKT/cyclin D1 cell cycle signaling via oxidative inactivation of PP2A in lowdose irradiated human fibroblasts

**DOI:** 10.18632/oncotarget.6518

**Published:** 2015-12-09

**Authors:** Tsutomu Shimura, Megumi Sasatani, Kenji Kamiya, Hidehiko Kawai, Yohei Inaba, Naoki Kunugita

**Affiliations:** ^1^ Department of Environmental Health, National Institute of Public Health, Saitama, Japan; ^2^ Department of Experimental Oncology, Research Institute for Radiation Biology and Medicine (RIRBM), Hiroshima University, Hiroshima, Japan; ^3^ Department of Molecular Radiobiology, Research Center for Radiation Genome Medicine, Research Institute for Radiation Biology and Medicine (RIRBM), Hiroshima University, Hiroshima, Japan

**Keywords:** mitochondria, ROS, cyclin D1, low-dose radiation, AKT

## Abstract

Here we investigated the cellular response of normal human fibroblasts to repeated exposure to low-dose radiation. In contrast to acute single radiation, low-dose fractionated radiation (FR) with 0.01 Gy/fraction or 0.05 Gy/fraction for 31 days increased in mitochondrial mass, decreased cellular levels of the antioxidant glutathione and caused persistent accumulation of mitochondrial reactive oxygen species (ROS). Excess ROS promoted oxidative inactivation of protein phosphatase PP2A which in turn led to disruption of normal negative feed-back control of AKT/cyclin D1 signaling in cells treated with long-term FR. The resulting abnormal nuclear accumulation of cyclin D1 causes growth retardation, cellular senescence and genome instability in low-dose irradiated cells. Thus, loss of redox control and subsequently elevated levels of ROS perturb signal transduction as a result of oxidative stress. Our study highlights a specific role of mitochondrial ROS in perturbation of AKT/cyclin D1 cell cycle signaling after low-dose long-term FR. The antioxidants N-acetyl-L-cysteine, TEMPO and mitochondrial-targeted antioxidant Mito-TEMPO provided protection against the harmful cell cycle perturbations induced by low-dose long-term FR.

## INTRODUCTION

Exposure to ionizing radiation (IR) induces reactive oxygen species (ROS) which affect intracellular metabolic redox control and perturb redox sensitive signaling pathways. Cysteine residues in proteins are a major ROS receptor and sense redox signals to generate sulfenic acid (R-SOH) or cysteine disulfide bonds (-S-S-) from cysteine thiols (R-SH) [1]. Such redox changes on specific proteins can result in altered conformation and/or activity, affecting signaling cascades and cell proliferation.

Mitochondria regulate ATP supply through oxidative phosphorylation. In addition to their pivotal role in cellular metabolism, mitochondria also play essential roles in stress responses and other physiological processes; for instance, mitochondria release cytochrome C to induce cell death [2, 3]. As part of their role in the regulation of signal transduction, mitochondria release ROS as second messengers for signaling pathways [4]. A certain amount of ROS is necessary for physiological processes such as differentiation, autophagy and adaptation to hypoxia [3, 5]. In contrast, higher ROS levels inflict oxidative damage to cellular components such as nucleic acids, proteins, and lipids, and inhibit cell proliferation. Mitochondrial ROS-mediated oxidative stresses are associated with induction of genomic instability in irradiated cells [6, 7]. However, the precise mechanisms underlying radiation-induced genome instability are not fully understood. In order to control ROS levels, mitochondria harbor antioxidant defense systems such as glutathione (GSH) and manganese superoxide dismutase (MnSOD) which scavenge ROS [8, 9]. Dismutation of superoxide anions in the mitochondria forms H_2_O_2_ either spontaneously or through the catalytic function of MnSOD in order to maintain the intracellular redox environment. Glutathione peroxidases then further reduce H_2_O_2_ to water by using glutathione as a ROS receptor.

AKT kinases are critical players in PI3K-mediated signal transduction pathways. In response to growth signals, AKT is activated downstream of PI3K and phosphorylates downstream substrates to transmit growth signals [10]. Active AKT mediates cell proliferation, cell survival, and cellular metabolism through multiple intracellular signaling pathways [11, 12]. DNA damage responses (DDR) have been well investigated using acute single radiation (SR) at high doses [13]. Following SR, AKT was shown to be transiently activated and then converted to an inactive dephosphorylated form 24 hours after irradiation [14]. AKT is dephosphorylated and inactivated by several phosphatases, including protein phosphatases 2A (PP2A), PH domain leucine-rich repeat protein phosphatase (PHLPP) and phosphatase with tensin homology (PTEN) [15–18]. DNA damage signaling generally stops when damaged DNA is repaired. In contrast to SR, DNA damage may accumulate with increasing cumulative radiation doses by repeated exposure, and the DDR may persist over a prolonged period. However, the cellular responses to long-term low doses of radiation remain uncertain due to lack of sufficient study. Recently, we have found that AKT activation persists over a prolonged period following long-term fractionated radiation (FR) for 31 days [14]. The radioresponse of AKT is changed from transient activation to constitutive activation according to duration of radiation exposure. PP2A, the phosphatase which is a negative regulator of AKT activity, is itself inactivated by oxidation of cysteine residues [19, 20]. Thus, ROS-mediated oxidative stresses might be involved in perturbations of the AKT signaling pathway via oxidative PP2A inactivation.

In this study, normal human fibroblasts were exposed to low doses (0.01Gy or 0.05Gy/fractions) of FR for 31 days. Low-dose long-term FR suppressed cell growth and induced cellular senescence with accumulation of mitochondrial ROS. Coincidently, PP2A activity was down-regulated in long-term FR cells. We found that long-term FR induced oxidative inactivation of PP2A via accumulation of mitochondrial ROS, thus leading to perturbations of the AKT pathway.

## RESULTS

### Increase in mitochondrial mass after low-dose long-term FR

**Figure 1:**
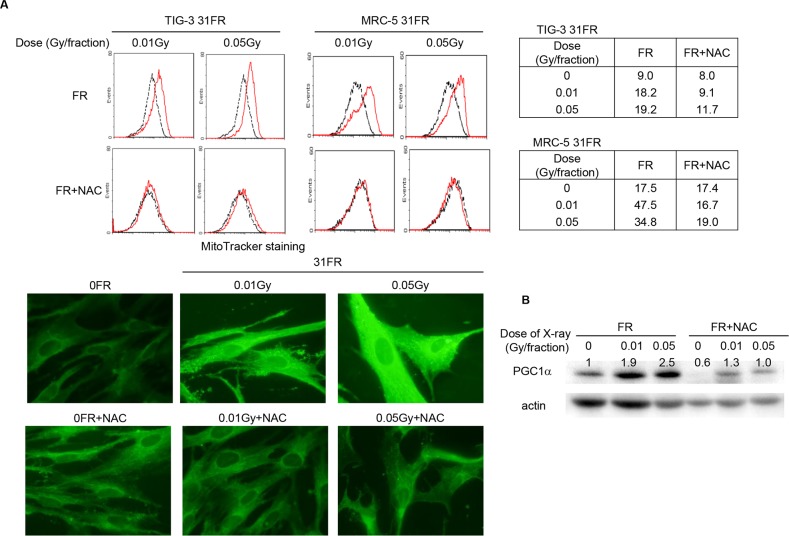
Mitochondrial mass, oxidative DNA damage in mitochondrial DNA and GSH levels in low-dose long-term FR cells **A.** Images of MitoTracker Grenn FM staining in untreated 0FR and 31FR cells of TIG-3 were shown on the lower panel. On the upper panel, FACS results for MitoTracker Green FM staining in untreated 0FR (dotted black lines) and 31FR cells (red lines). Mean fluorescence intensity values of the MitoTracker staining were shown on the upper right panel. **B.** Western blotting of PGC1α and actin was shown in 0FR and 31FR cells with and without NAC. The amounts of PGC1α were normalized by corresponding actin level. The values are expressed relative to the control value of 0FR cells.

We exposed MRC-5 and TIG-3 normal human fibroblast cells to 0.01- or 0.05-Gy fractions of X-rays. Cells exposed to FR for 31 days were referred to as 31FR cells. The morphological observations of mitochondria and FACS analysis with MitoTracker Green FM staining revealed that 31FR MRC-5 and 31FR TIG-3 cells exhibited an increase in mitochondrial mass compared to unirradiated 0FR cells (Figure 1A). The expression of PPAR-γ co-activator-1 α (PGC1-α) which stimulates mitochondrial biogenesis was examined in 31FR TIG-3 cells by western blotting (Figure 1B). Consistent with the result of increase in mitochondrial mass, the PGC1-α expression elevated after low-dose long-term FR in 31FR cells. Cells were continuously treated with N-acetyl-L-cysteine (NAC) to mitigate oxidative stresses at a final concentration of 1 mM during FR exposure. Medium changes were made at 2- or 3-days intervals. Continuous NAC treatment prevented increase in mitochondrial mass and induction of PGC1-α expression in 31FR cells (Figure 1A, 1B).

### Accumulation of mitochondrial ROS in low-dose FR cells

**Figure 2:**
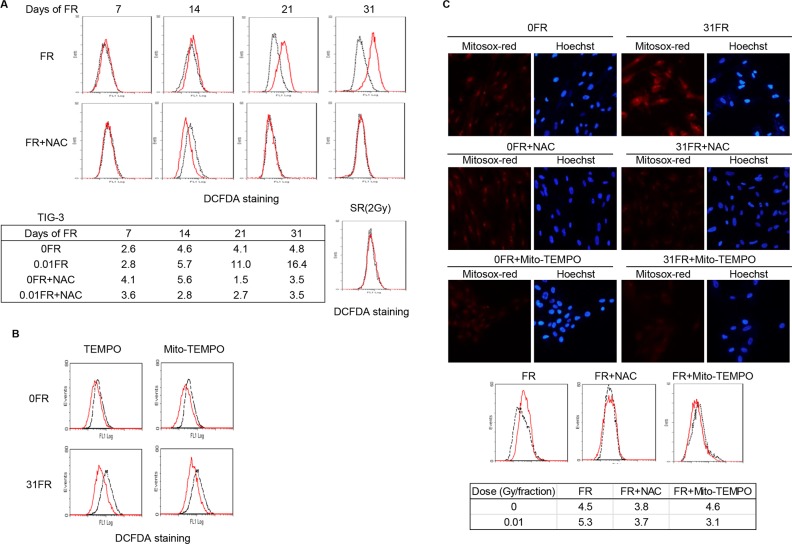
ROS generation in 0FR and 31FR cells **A.** FACS results for DCFDA staining in untreated (dotted black lines) and treated TIG-3 cells (red lines). Mean fluorescence intensity values of DCFDA staining were shown. **B.** FACS results for DCFDA staining in untreated 0FR and 31FR cells (dotted black lines) and TEMPO- or Mito-TEMPO-treated TIG-3 0FR and 31FR cells (red lines). **C.** Images of Mitosox-red staining cells in control 0FR and 31FR cells of TIG-3 with and without NAC or Mito-TEMPO treatment. Mean fluorescence intensity values of Mitosox-red staining were shown.

ROS are produced in mitochondria as a by-product of ATP production through oxidative phosphorylation. Therefore, we investigated the effect of increase in mitochondrial mass on generation of ROS after long-term FR. We measured the amounts of ROS in cells by staining them with 2′,7′-dichlorofluorescin diacetate(DCFD) 24 hours after last FR at indicated days (Figure 2A). Distribution of DCFDA-stained cells was unchanged between unirradiated cells and FR cells until 14 days, while strong DCFDA-positive cells appeared when cells were exposed to FR for > 21 days and were further increased at 31 days (Figure 2A, upper left panel, FR). In contrast, ROS accumulation was not observed in TIG-3 cells exposed to 2 Gy of SR (Figure 2A, upper right panel, SR). ROS were also not induced in TIG-3 cells cultured for 31 days without irradiation (Figure 2A, dotted black line, upper left panel, FR). Similarly, induction of ROS was observed after low-dose long-term FR in MRC-5 cells when cells were exposed to FR for > 21 days but not after SR (Figure S1A). Induction of ROS was not observed by continuous NAC treatment in MRC-5 and TIG-3 cells regardless of FR exposure (Figure 2A, red line, lower right panel, FR+NAC and Figure S1A). Furthermore, ROS accumulation was suppressed by treatment with other antioxidants 2,2,6,6-Tetramethylpiperidine 1-Oxyl (TEMPO) or (2-(2,2,6,6-Tetramethylpiperidin-1-oxyl-4-ylamino)-2-oxoethyl)triphenylphosphonium chloride (Mito-TEMPO) for 24 hours in TIG-3 31FR cells (Figure 2B).

Mitochondrial ROS were stained with MitoSOX-red which is a fluorogenic dye for highly selective detection of superoxide in mitochondria. Strong intensity of MitoSOX-red staining was evident in 31FR TIG-3 cells treated with 0.01Gy/fraction, but not in unirradiated control cells (Figure 2C upper panel). MitoSOX-red positive cells were also evident in 31FR MRC-5 cells (Figure S1B). NAC and Mito-TEMPO treatment eliminated MitoSOX-red staining in 31FR cells (Figure 2C middle panel and lower panel). Thus, mitochondrial ROS are generated in 31FR cells with increase in mitochondrial mass (Figure 1).

### Oxidative mitochondrial DNA damage and decrease in GSH levels after low-dose long-term FR

**Figure 3:**
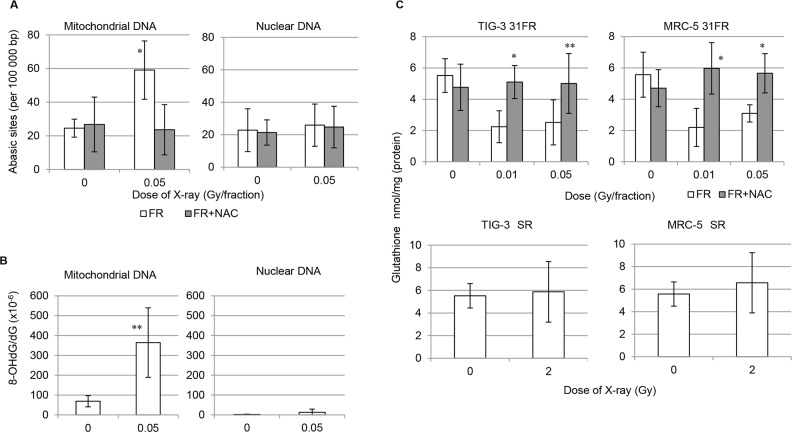
Mitochondrial DNA damage and GSH levels in 0FR and 31FR cells **A.** The number of abasic sites was measured by the DNA damage quantification assay performed on nuclear and mitochondrial DNA in control 0FR and 31FR cells of TIG-3 with and without NAC treatment. **B.** 8-OHdG levels on nuclear and mitochondrial DNA in control 0FR and 31FR cells of TIG-3. **C.** Cellular GSH levels were shown for control and 31FR TIG-3 cells in the upper left panel and for control and 31FR-treated MRC-5 cells in the upper right panel. Cellular GSH levels were shown in unirradiated cells and at 24 hours after 2-Gy irradiation for TIG-3 cells in the lower left panel and for MRC-5 in the lower right panel.

We next quantified oxidative damage on nuclear and mitochondrial DNA after low-dose long-term FR by measuring amounts of apurinic/apyrimidinic (AP) site using Nucleostain DNA damage Quantification Kit. As shown in Figure 3A on left panel, the number of AP-sites in mitochondrial DNA greatly increased in 31FR cells compared to unirradiated 0FR cells. Thus, oxidative mitochondrial DNA damage persisted at least for 24 hours following low-dose long-term FR. Accumulation of AP sites in nuclear DNA was not observed among all four samples regardless of treatment (Figure 3A, right panel). These results indicated that mitochondrial DNA is more sensitive to oxidative stresses triggered by low-dose long-term FR than nuclear DNA in human cells. Accumulation of oxidative mitochondrial DNA damage was suppressed by continuous NAC treatment in 31FR MRC-5 and 31FR TIG-3 cells (Figure 3A). We further evaluated oxidative DNA damage in nuclear and mitochondrial DNA after low-dose long-term FR by measuring amounts of 8-Hydroxydeoxyguanosine (8-OHdG) using high performance liquid chromatographyelectrochemicaldetector (HPLC-ECD). As shown in Figure 3B, 8-OHdG accumulated in the mitochondrial DNA of TIG-3 31FR cells compared to that in unirradiated TIG-3 0FR cells. In contrast, 8-OHdG levels were low in nuclear DNA of 0FR and 31FR cells of TIG-3 regardless of FR exposure.

We next investigated the effect of long-term FR on antioxidant defense systems against mitochondrial ROS. Amounts of the cellular antioxidant GSH were measured in unirradiated control 0FR cells and 31FR cells. GSH levels were unchanged at 24 hours after 2 Gy of SR in MRC-5 and TIG-3 cells (Figure 3C, lower panel). In contrast, GSH levels decreased in MRC-5 31FR cells and TIG-3 31FR cells compared to that of unirradiated control cells (Figure 3C, upper panel). Decrease in GSH levels after low-dose long-term FR was not observed in continuous NAC-treated 31FR cells.

### Perturbation of AKT signaling via down-regulation of PP2A activity after long-term FR

**Figure 4:**
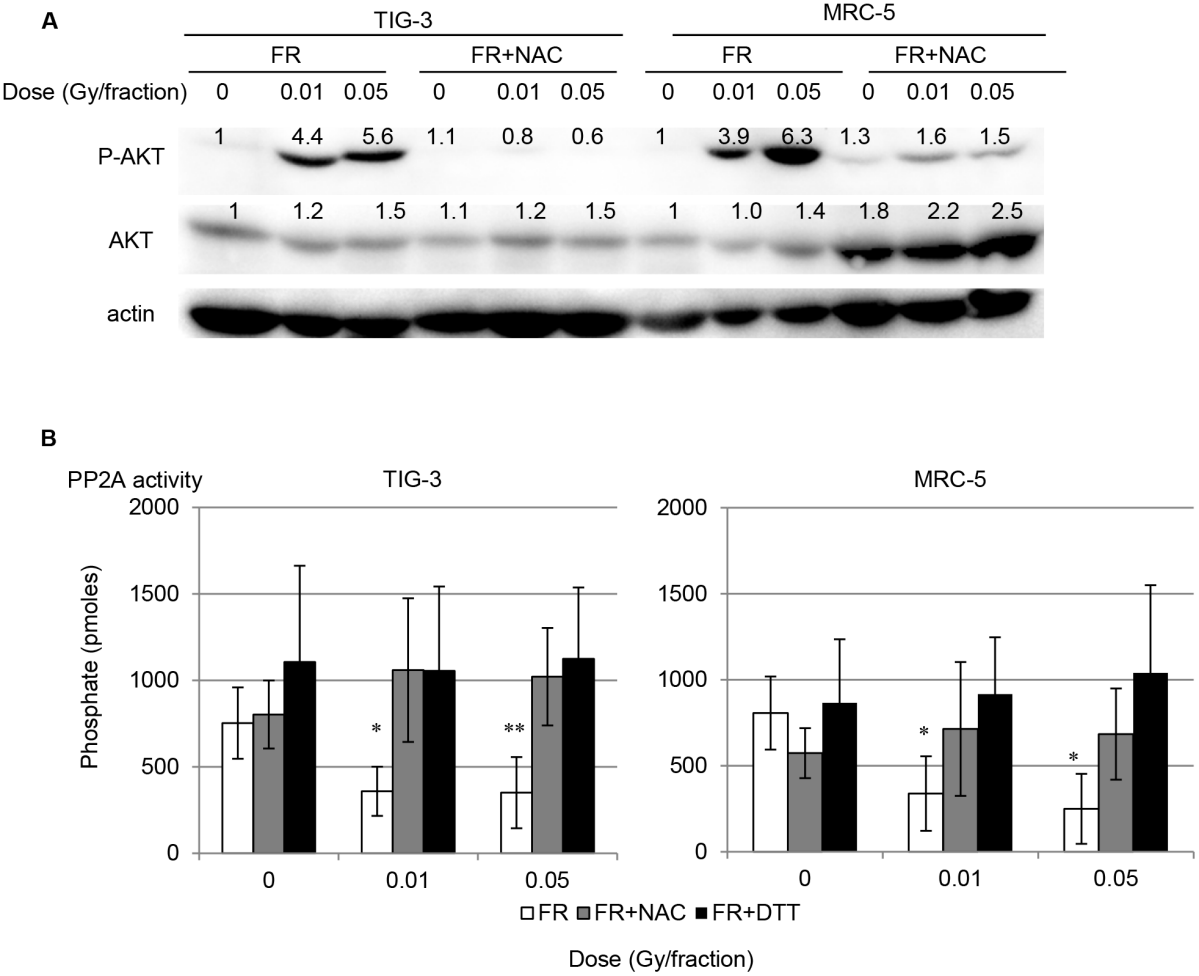
AKT activation after long-term FR AKT activation via down-regulation of PP2A activity after low-dose long-term FR **A.** AKT phosphorylation on Ser473 in TIG-3 31FR ells and MRC-5 31FR cells with and without NAC treatment. The amounts of total AKT and p-AKT were normalized by corresponding actin level. The values are expressed relative to the control value of 0FR cells. **B.** PP2A activity in unirradiated controls and cells treated with 31 days of FR (0.01 Gy/fraction and 0.05 Gy/fraction) with and without NAC. For DTT-treated samples, DTT (1 mM) was added to the lysis buffer.

ROS oxidize phosphatases and modulate cellular signaling pathways [21]. The effect of ROS accumulation on AKT signaling was investigated by examination of phosphorylation of AKT on serine 473 in FR-treated MRC-5 cells. Consistent with our report in other cell types [14], AKT in MRC-5 cells was transiently activated by 2 Gy of SR, with levels of active AKT declining to control levels by 24 hours (Figure S2). AKT activation was evident at least 24 hours after the last FR treatment when cells were exposed to 0.01 Gy and 0.05 Gy of FR for 31 days, consistent with the timing of ROS accumulation (Figure 4A). Continuous NAC treatment suppressed AKT activation in MRC-5 31FR cells (Figure 4A). These results suggest that accumulation of mitochondrial ROS affects AKT activation after long-term FR.

The protein phosphatase PP2A plays a key role in controlling AKT inactivation through its dephosphorylation of AKT on both Serine 473 and Threonine 308 [18]. We measured PP2A activity after low-dose long-term FR. PP2A activity was attenuated in 31FR MRC-5 and 31FR TIG-3 cells compared to that of control cells (Figure 4B). NAC treatment prevented down-regulation of PP2A activity in 31FR cells (Figure 4B). PP2A activity was restored by DTT treatment to control levels (Figure 4B).

**Figure 5:**
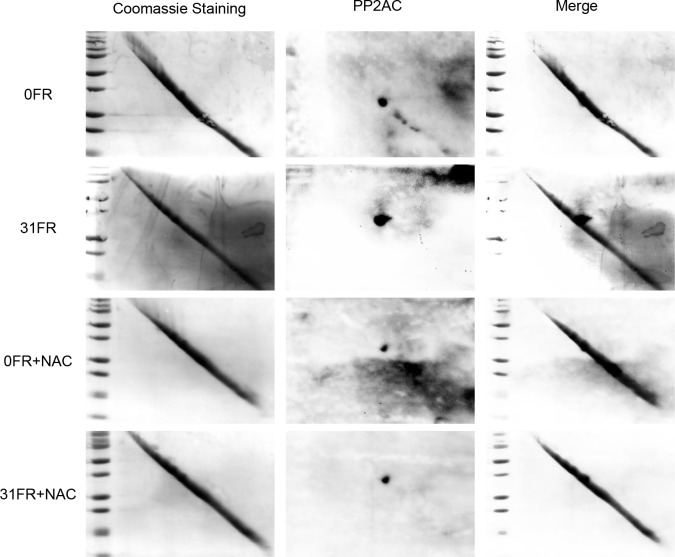
Disulfide modification of PP2A Cell lysates were collected from TIG-3 cells at 24 hours after the last FR treatment Proteins separated by diagonal electrophoresis were stained with Coomassie blue (left panel). Western blotting of PP2AC was shown on the right panel in 0FR and 31FR cells with and without NAC.

Protein phosphatases are known to be susceptible to a number of redox-dependent modifications, including an interchange between the reduced thiol and several different oxidized disulfide states. Thus, we hypothesized that in cells subjected to long-term FR, ROS may oxidize PP2A on its active-site cysteine, thus inactivating PP2A. Disulfide bond formation in PP2Ac was examined by diagonal electrophoresis with western blotting (Figure 5). PP2A with an intrarmolecular disulfide (above the diagonal) was detected in 31FR cells but not in 0FR cells. NAC treatment prevented disulfide bond formation of PP2A in 31FR cells (Figure 5).

### Abnormal nuclear cyclin D1 accumulation in long-term FR cells

**Figure 6:**
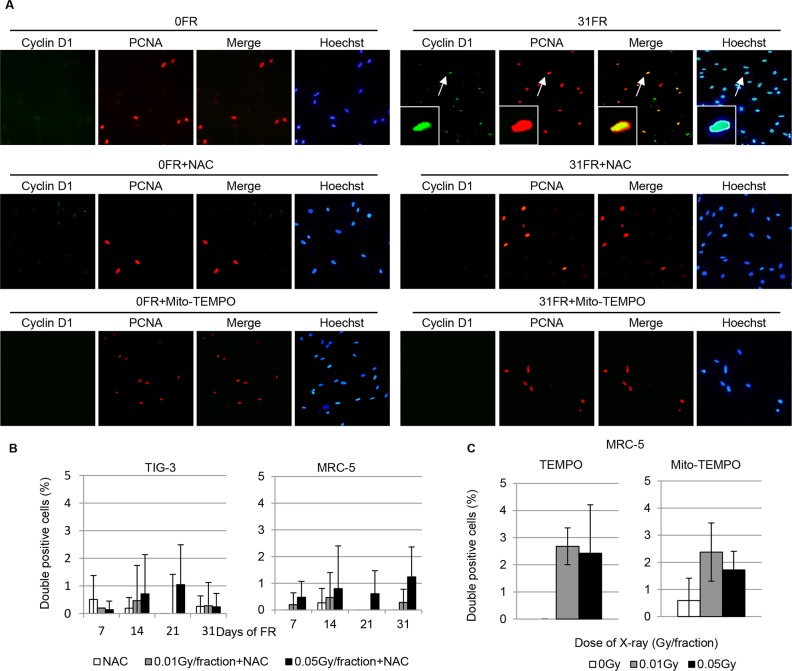
Inhibition of nuclear cyclin D1 accumulation by NAC in 31FR cells **A.** Immunofluorescence localization of cyclin D1 and PCNA is shown for control and 31FR MRC-5 cells with and without NAC treatment. DNA was stained with Hoechst. Magnified images are inserted. **B.** Cells double positive for cyclin D1 and PCNA were scored 24 hours following the indicated FR exposure. Data for MRC-5 and TIG-3 cells exposed to 0.01-Gy or 0.05-Gy fractions plus NAC are shown.

We have previously reported that perturbation of AKT signaling causes persisted nuclear cyclin D1 expression during DNA replication in normal human fibroblasts [22]. To detect abnormal nuclear accumulation of cyclin D1 in S-phase, cells were treated with a hypotonic buffer containing detergent to remove cytoplasmic cyclin D1. Proliferating cell nuclear antigen (PCNA) immunofluorescence was used to identify cells in S phase [22]. Detergent-insoluble nuclear cyclin D1 was detected in PCNA-positive nuclei of cells exposed to 0.05 Gy of FR for 31 days (Figure 6A upper right panel). We have previously reported that in 31FR MRC-5 and TIG-3 cells, the percentage of cyclin D1 and PCNA double positive cells is about 30% [22]. Thus, perturbation of AKT signaling after long-term FR would be expected to result in abnormalities in cell cycle control. Treatment with antioxidants such as NAC, TEMPO or Mito-TEMPO suppressed induction of abnormal nuclear cyclin D1 in 31FR cells (Figure 6A, 6B and 6C).

### Growth suppression and induction of cellular senescence after low-dose FR

**Figure 7:**
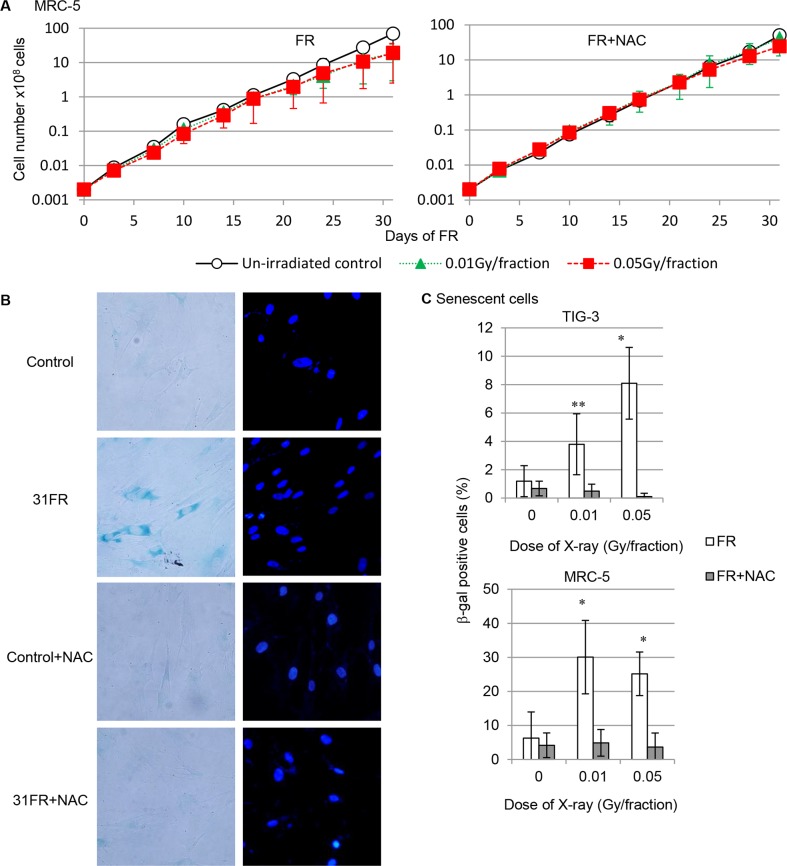
Growth retardation and cellular senescence after low-dose long-term FR **A.** Cell growth of unirradiated cells (open circles) and cells exposed to 0.01-Gy (open triangles) and 0.05-Gy (open squares) fractions. Growth curves for MRC-5 cells with and without NAC treatment are shown. **B.** Images of β-gal staining in unirradiated control and irradiated 31FR cells of MRC-5 with/without NAC treatment. **C.** Percentage of β-gal staining cells in unirradiated control and irradiated 31FR cells with/without NAC treatment.

We next analyzed the cell growth of FR-treated cells after administration of the antioxidant NAC. Exposure to low-dose FR decelerated cell proliferation in both cell lines as we reported previously (Figure 7A left panel, Figure S3) [22]. NAC was shown to rescue growth retardation induced by low-dose long-term FR (Figure 7A right panel, Figure S3).

Cellular senescence is thought to be induced by ROS-mediated oxidative stress after long-term FR. Therefore, we investigated cellular senescence in response to FR. Senescent cells were identified by detection of senescence-associated β-galactosidase (β-gal) activity. Figure 7B depicts representative β-gal staining in MRC-5 cells irradiated with 0.05 Gy/fraction for 31 days. The percentage of β-gal-positive senescent cells increased remarkably in both MRC-5 and TIG-3 cells treated with FR for 31 days compared to cells cultured for 31 days without FR (Figure 7C). PML bodies, a marker for senescent cells were evident in 31FR cells but not in 0FR cells (Figure S4). Thus, growth retardation induced by low-dose long-term FR was partially due to an increase in senescent cells. Treatment with the antioxidant NAC eliminated this FR-induced increase in senescence. (Figure 7B, 7C). Our results indicate that oxidative stress mediates growth retardation and cellular senescence of MRC-5 and TIG-3 cells after low-dose long-term FR.

## DISCUSSION

Here, we demonstrate that low-dose long-term FR causes increase in mitochondrial mass and persistently increased levels of mitochondrial ROS in normal human cells. Mitochondria express the antioxidant GSH to protect cells against oxygen toxicity [9]. Cellular GSH levels are decreased by GSH consumption and lost through efflux in response to oxidative stress and many pathological conditions [23]. We here demonstrated that cellular GSH was decreased by low-dose long-term FR. Insufficient GSH levels lead to a decrease in antioxidant capacity, accumulation of mitochondrial ROS, and stimulation of long-lasting oxidative stresses in long-term FR cells. NAC serves as a precursor of cysteine and augments intracellular levels of GSH [24]. Therefore, oxidative stresses after long-term FR were reversed by administration of NAC. GSH deficiency is associated with increased hemolysis, cataracts and central nervous system abnormalities [9]. Interestingly, GSH depletion and mitochondrial dysfunction are induced in response to very low-dose chronic exposure to persistent organic pollutants such as 2,3,7,8-Tetrachlorodibenzo-*p*-dioxin [25, 26].

Here we found that AKT activation was persistent in normal human fibroblasts treated with long-term FR, but not in cells treated with 2 Gy of SR. Coincidently, mitochondrial ROS accumulated in these long-term FR cells. Therefore, persistent oxidative stresses may affect AKT signaling after long-term FR. AKT inactivation is mediated by dephosphorylation of the protein via several phosphatases including PP2A [18]. However, ROS oxidizes PP2A on cysteine residues and down-regulates PP2A activity in long-term FR cells. This loss of PP2A activity can thus lead to a loss of negative feedback control of the AKT pathway, leading to persistent, long-lasting AKT activity in cells after long-term FR.

We here demonstrated that mitochondrial ROS perturbed the AKT/cyclin D1 pathway after long-term FR. Perturbation of AKT signaling and cyclin D1 expression is shown to be implicated in tumoregenesis due to inappropriate cell cycle entry [10, 27, 28]. Aberrant cyclin D1 expression provides a driving force behind the development of tumorigenesis and is often detected in premalignant and malignant tissues. Abnormal nuclear cyclin D1 accumulation in S phase induces DNA replication stresses and resulting DNA double strand breaks, and is associated with induction of genomic instability in irradiated cells [22, 29]. Perturbation of cyclin D1 expression also associates with cellular senescence [30, 31]. Data from this current study indicates that induction of nuclear cyclin D1 accumulation was suppressed by treatment with antioxidants NAC, TEMPO and Mito-TEMPO in 31FR MRC-5 and TIG-3 cells. Thus, perturbation of the AKT/cyclin D1 pathway after low-dose long-term FR is attributable to ROS-mediated oxidative stress. Increasing antioxidant capacity is critical to preventing abnormalities in cyclin D1 expression after long-term FR.

In conclusion, we have demonstrated a link between mitochondrial dysfunction and perturbation of the AKT/cyclin D1 cell cycle signaling in low-dose irradiated human cells. Mitochondria are the target organelle for low-dose radiation. Administration of antioxidants may be effective in mitigating the toxicity of low-dose long-term FR in order to guard genome stability in irradiated cells. Our findings will provide new insights in cancer risk estimation associated with long-term low-dose radiation exposure.

## METHODS

### Cell culture conditions and drugs

Normal human diploid lung fibroblasts (MRC-5 and TIG-3) were purchased from the Health Science Research Resources Bank (Osaka, Japan), and grown in minimum essential medium (Nacalai Tesque, Kyoto, Japan) supplemented with 10% heat-inactivated fetal calf serum. Serial passage experiments were started after 33 or 23 population doublings for MRC-5 and TIG-3 cells, respectively. NAC and Mito-TEMPO were purchased from Sigma (San Diego, CA). TEMPO was purchased from Tokyo Chemical Industry Co., Ltd (Tokyo, Japan). Cells were treated with TEMPO or Mito-TEMPO at a final concentration of 100 μM for 24 hours.

### Irradiation experiments

Cells were irradiated using a 150-kVp X-ray generator (Model MBR-1505R2, Hitachi, Tokyo, Japan) with a 0.5-mm Cu and 0.1-mm Al filter at a dose of 0.7 Gy/min. Low dose X-ray fractions (0.01 or 0.05 Gy) were administrated twice a day and 5 days/week. Total doses delivered over 31 days were 0.46 Gy and 2.3 Gy for cells exposed to FR of 0.01 Gy and 0.05 Gy, respectively.

### ROS detection and mitochondrial mass measurement

Cells were stained with 20 μM DCFDA (Sigma) or 400nM MitoTracker Green FM (invitrogen) in minimum essential medium without serum for 30 min at 24 h after the last FR at indicated days. DCFDA-, MitoSOX-red- or MitoTracker Green FM-stained cells were quantified with a FACScan (Becton Dickinson, USA). Cells were placed on glass slides and cultured overnight. Cells on coverslips were stained with MitoSOX-red or MitoTracker Green FM according to the manufacturer's instructions (Invitrogen, Carlsbad, CA). Images were acquired using a CCD camera attached to a fluorescence microscope (Keyence, Osaka, Japan).

### Immunofluorescence

Immunofluorescence stainings were performed as described [22, 32]. For PML immunostaining, cells were fixed with 4% formaldehyde for 10 min and permeabilized with 0.25% Triton X-100 for 5 min. Antibodies against PML (Milipore, MA), PCNA (Santa Cruz Biotechnology, Santa Cruz, CA) and cyclin D1 (Nichirei Bioscience, Tokyo, Japan) and secondary antibodies conjugated with Alexa Fluor 488 (Molecular Probes, Eugene, OR) or Cy-3 (Jackson ImmunoResearch Laboratories, West Grove, PA) were used. Cells were counterstained for DNA with Hoechst 33258 (4 μg/mL in Vectashield mounting medium; Vector Laboratories, Burlingame, CA). Images were captured using a CCD camera attached to a fluorescence microscope (Keyence, Osaka, Japan). For each data point, > 50 cells were counted from at least three independent samples.

### Western blot analyses

Western blotting was performed as described [14]. Primary antibodies against β-actin (A2066, Sigma), AKT (Cell Signaling, Beverly, MA, USA), phospho-AKT-Ser473 (Cell Signaling), PP2AC (Cell Signaling) and a secondary goat anti-rabbit antibody conjugated with horseradish peroxidase (GE Healthcare, Little Chalfont, UK) were used. Protein bands were visualized using Chemi-Lumi One L Western blotting substrate (Nacalai Tesque), and band intensities were measured using Image Lab software (Bio-Rad).

### Measurement of GSH

Total GSH was quantified using a Total Glutathione Quantification Kit (Dojindo) according to the manufacturer's protocol. OD values at 405 nm were measured with a microplate reader (Sunrise).

### Measurement of PP2A phosphatase activity

PP2A phosphatase activity was measured from cell lysates (100 μg) using a PP2A immunoprecipitation phosphatase assay kit according to the manufacturer's instructions (Millipore, Temecula, CA). DTT (1mM) was added to the lysis buffer for DTT-treated samples.

### Analysis of oxidative DNA damage in nuclear and mitochondria DNA

Mitochondrial DNA and nuclear DNA were isolated by using a mitochondrial DNA isolation kit (Biovision) and a DNA Extraction WB kit (Wako Pure Chemical Industries, Osaka Japan), respectively. The isolated DNA was labeled with N-Amino-oxymethylcarbonyl-hydrazino-D-biotin by the manufacturer's instructions (Nucleostain DNA damage Quantification Kit; Dojindo). OD values at 650 nm were measured with a microplate reader (Sunrise). For 8-OHdG Assay, the isolated DNA was digested with nuclease P1 followed by the manufacturer's instructions (8-OHdG Assay Preparation reagent set; Wako Pure Chemical Industries). The amounts of 8-OHdG were quantified using high performance liquid chromatography-electrochemical detector (HPLC-ECD) as described previously [33].

### Diagonal electrophoresis

Diagonal electrophoresis was carried out as followed by Moreno et.al. [34]. First dimension is performed under nonreducing conditions and second dimension under reducing conditions. Proteins with intermolecular disulfide bridges appear below the diagonal, while proteins with intramolecular disulfide bridges appear above the diagonal.

### Cell growth assay

Cells (2 × 10^5^) were seeded into 25-cm^2^ flasks (Thermo Fisher Scientific, Waltham, MA), incubated overnight, and irradiated daily. Growth rates were monitored by counting cell numbers twice a week. When the total cell number exceeded 2 × 10^5^, cells were subcultured to 2 × 10^5^ cells in a new flask.

### Senescence

Cellular senescence was examined by using the Senescence Detection Kit (BioVision) according to the manufacturer's instructions. Images were captured using a CCD camera attached to a fluorescence microscope (Keyence, Osaka, Japan). For each data point > 50 cells were counted from at least three independent samples.

### Statistical analysis

Error bars represent standard deviations. All experiments were repeated at least three times using independent samples. Student's *t*-test was used for statistical analyses. Single and double asterisks indicate significant differences at *p* < 0.01 and *p* < 0.05, respectively.

## SUPPLEMENTARY FIGURES


