# Supplementary material for: Mitochondrial reactive oxygen species perturb AKT/cyclin D1 cell cycle signaling via oxidative inactivation of PP2A in lowdose irradiated human fibroblasts
Source: Oncotarget. 2015 Dec 9;7(3):3559–70. doi: 10.18632/oncotarget.6518 (PMC4823127; doi:10.18632/oncotarget.6518)
Supplement: Supplementary file 1 [file oncotarget-07-3559-s001.pdf]

SUPPLEMENTARY FIGURES

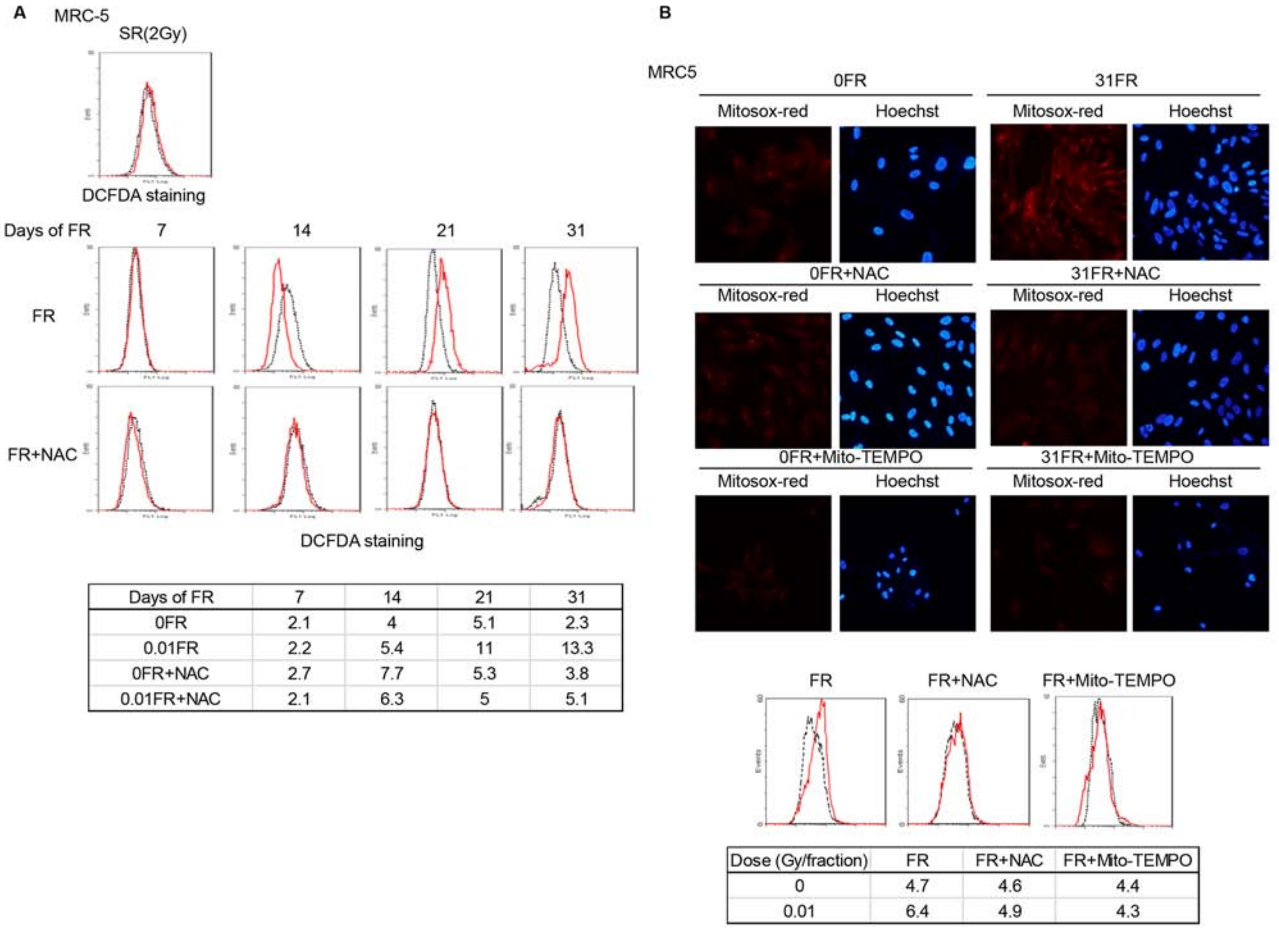

Days of FR	7	14	21	31
0FR	2.1	4	5.1	2.3
0.01FR	2.2	5.4	11	13.3
0FR+NAC	2.7	7.7	5.3	3.8
0.01FR+NAC	2.1	6.3	5	5.1

Dose (Gy/fraction)	FR	FR+NAC	FR+Mito-TEMPO
0	4.7	4.6	4.4
0.01	6.4	4.9	4.3

Supplementary Figure S1: ROS generation in 0FR and 31FR MCR-5 cells. A. FACS results for DCFDA staining in untreated (dotted black lines) and treated MRC-5 cells (red lines). Mean fluorescence intensity values of DCFDA staining were shown. **B.** Images of Mitosox-red staining cells in control 0FR and 31FR cells of TIG-3 with and without NAC or Mito-TEMPO treatment. Mean fluorescence intensity values of Mitosox-red staining were shown.

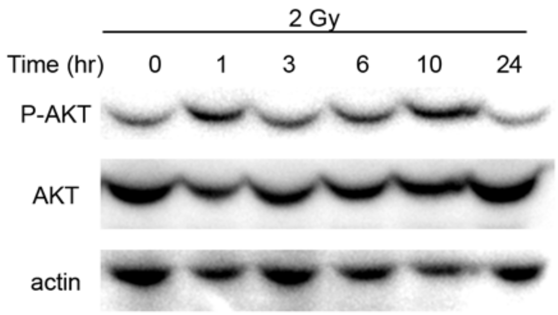

Supplementary Figure S2: AKT activation after SR. AKT phosphorylation on Ser473 (P-AKTSer473, active AKT) after 2Gy of SR exposure in MRC-5 cells.

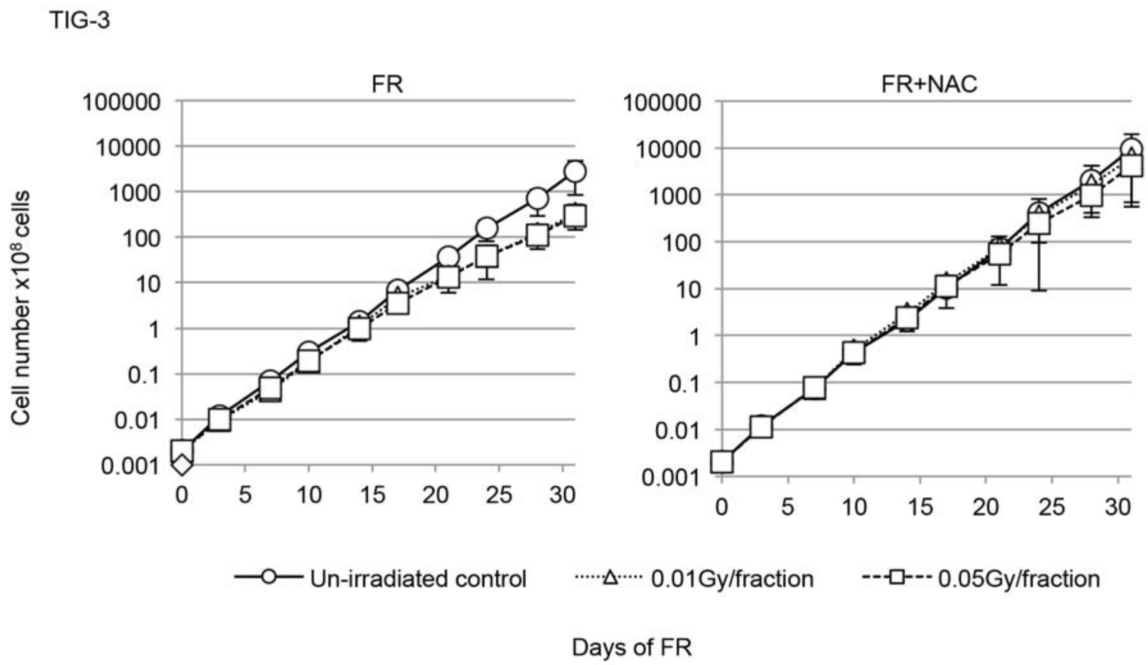

Supplementary Figure S3: Growth retardation of TIG-3 cells after low-dose long-term FR. Cell growth of unirradiated cells (open circles) and cells exposed to 0.01-Gy (open triangles) and 0.05-Gy (open squares) fractions. Growth curves for TIG-3 cells are shown.

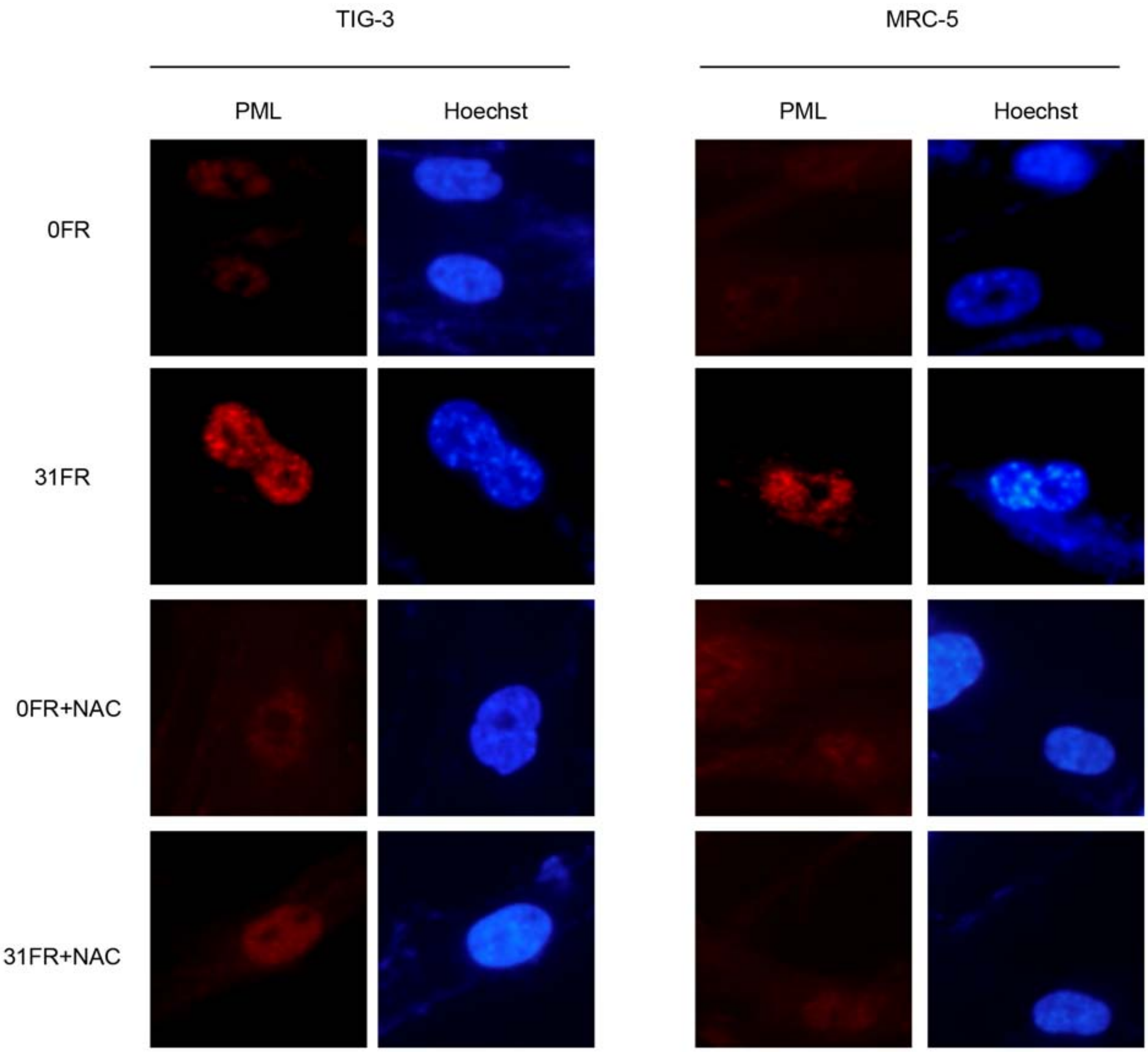

Supplementary Figure S4: Induction of PML nuclear bodies after low-dose long-term FR. Immunofluorescence staining with anti-PML antibody was shown in unirradiated control and irradiated 31FR cells of MRC-5 with/without NAC treatment.
